# A fence barrier method of leading edge cell capture for explorative biochemical research

**DOI:** 10.1080/19336918.2016.1269997

**Published:** 2017-02-17

**Authors:** Lucas J. Wager, Rachael Z. Murray, Erik W. Thompson, David I. Leavesley

**Affiliations:** a Wound Management Innovation Cooperative Research Centre, Australia; b Institute of Health and Biomedical Innovation and School of Biomedical Sciences, Queensland University of Technology, Australia; c Translational Research Institute, Brisbane, Australia; d Institute of Medical Biology, Agency for Science, Technology and Research (A*STAR), Singapore

**Keywords:** cell migration, collective migration, leading edge, migration assay, wound healing

## Abstract

The scratch or wound-healing assay is used ubiquitously for investigating re-epithelialisation and has already revealed the importance of cells comprising the leading edge of healing epithelial wounds. However it is currently limited to studying the effect of known biochemical agents on the tissue of choice. Here we present an adaptation that extends the utility of this model to encompass the collection of cells from the leading edge of migrating epithelial sheets making available explorative biochemical analyses. The method is scalable and does not require expensive apparatus, making it suitable for large and small laboratories alike. We detail the application of our method and exemplify proof of principle data derived from primary human keratinocyte cultures.

## Introduction

Re-epithelialisation of the epidermis after injury is arguably the most common and crucial form of collective cell migration that occurs during adult life. In cutaneous wound closure, re-epithelialisation begins after disruption to the confluent collection of keratinocytes that form the epidermis. This entails a cascade of biological processes that culminate in the migration of keratinocytes at the wound-edge into and over the wound bed.^1^ It is important that wound closure is completed quickly to prevent dehydration, reduce microbial infection and restore homeostasis. Furthermore, delays to cutaneous wound healing correlate with increased scarring and in severe cases chronic non-healing wounds that can lead to limb amputation.^2-4^ A recent and conservative estimate of the burden of treating non-healing wounds in the UK's National Health Service places the cost at £3 billion annually.^5^ The cost burden of chronic wounds in Australia's health system (in 2012) was estimated to be US$2.85 billion annually.^6^ Thus it is clear that research aimed at improving wound healing is well founded and has led to the development of numerous laboratory models of wound healing for this purpose.

In vitro models of 2D cell migration are used extensively to investigate the biochemical processes of cell migration, primarily by employing the ubiquitous ‘scratch assay’.^7^ In the scratch assay, an area of cell exclusion is created in a confluent monolayer by ‘scratching’ cells away, most commonly with a pipette-tip. This technique in many ways recapitulates cutaneous wound re-epithelialisation, and as such is commonly referred to as a ‘wound-healing assay’. In practice, cell migration into the ‘scratch’ or ‘wound’ is then quantified over time, and often measured in parallel to cells cultured in augmented growth media or over substrate-coated surfaces for comparison. The scratch wound is thus simple and inexpensive to implement, in stark contrast to the inherently complex study of re-epithelialisation using in vivo models.

While in vitro models are indeed a dramatic simplification of wound healing in vivo they nonetheless capture many aspects of wound re-epithelialisation. During re-epithelialisation, cells at the leading edge exhibit obvious responses to stimuli and contributions to sheet migration, undergoing dramatic cytoskeletal changes: extending lamellipodia, filopodia and polarizing in the direction of migration.^8^ Furthermore, actin stress fibers and ‘purse strings’ are evident at the leading edge of some wound assays.^8^ In this vein, even individual cells can take on a commanding role in collective migration, forming a subclass of cells termed ‘leader cells’ that have a specific phenotype and gene expression independent of the cell collective.^9,10^ Underlying all of these apparent morphological changes at the leading edge of migrating sheets are less obvious changes occurring at a molecular level. For example, extracellular matrix (ECM) deposition is altered and in turn, so too are receptors that interact with the ECM such as integrins.^11,12^ Cell adhesion and cell cohesion molecules are also altered, evidenced by changes to the function of desmosomal protein complexes.^13^ Importantly, such diversification at the leading edge evidenced in vivo is clinically relevant and is reflected in the margins of non-healing wounds of patients.^13^

The subpopulations of cells at the leading edge of migrating epithelial sheets and, in turn re-epithelialisation, are an important target to study for their leading role in wound healing. However, a notable convenience of in vitro study that has not evolved in scratch assay models is an ability to separate sub-populations of cells that comprise the healing monolayer after wounding. As it is, few techniques are available to resolve and explore the biochemistry of such cellular subsets. One such technique is laser-assisted microdissection (LAM). While LAM provides unmatched resolution as to which cells can be captured and studied, it requires access to specialist equipment, is a lengthy procedure, and returns only small numbers of cells. These aspects make the accumulation of adequate sample sizes for extensive biochemical analysis, such as RNA-seq, a challenging undertaking. Another approach is to create a large number of ‘cell islands’ sufficiently small to induce a wound healing response in all cells.^14,15^ While this method is relatively less expensive than LAM it still requires the purchase or engineering of a large apparatus designed specifically for wounding of cell monolayers.

**Figure 1.**
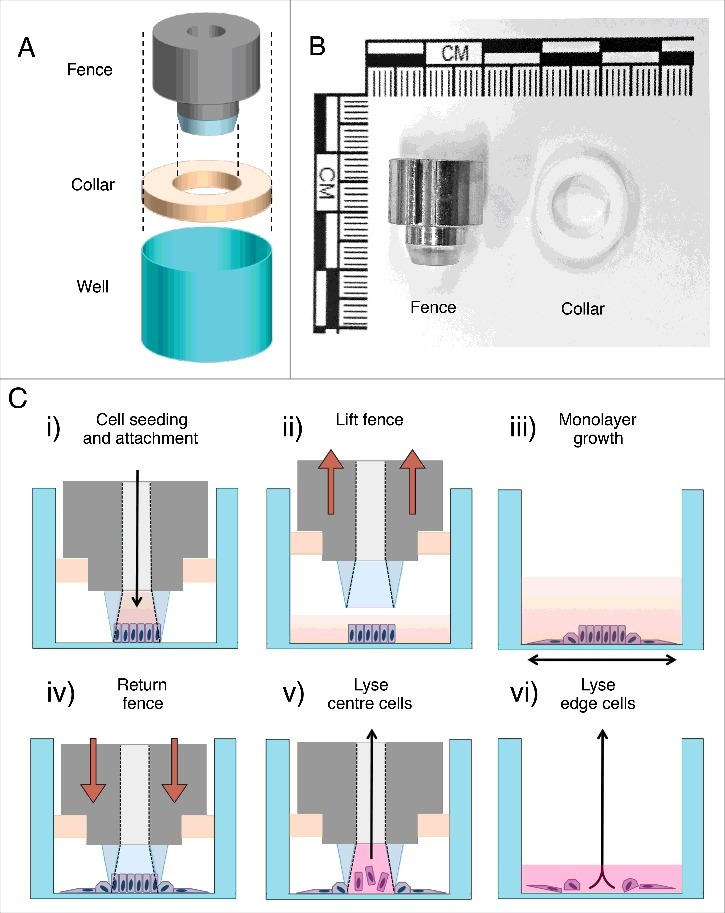
(A) A schematic illustrating the components of the fence and collar system relative to a representative well from a 24-well tissue culture plate. (B) A photograph of the fence (left) and collar (right). (C) A flow diagram illustrating the application of the fence and collar. (C, i) The fence is inserted into the collar and the fence-collar unit is inserted into the tissue culture well, forming an inner-well. Cells are seeded into this inner-well. C ii) After cells have attached, the fence-collar unit is removed. C iii) The monolayer is washed then media is added to the well for incubation. C iv) After incubation the monolayer is washed and the monolayer treated with RNAlater preservation reagent before the fence-collar unit is returned to the well, reproducing the inner-well. C v) TRIzol is added to the inner chamber and the cells are lysed by repeatedly pipetting the volume up and down. The TRIzol-cell lysate is then aspirated and retained as the ‘center’ sample. C vi) The fence-collar unit is removed and TRIzol is added to the culture plate well. The remaining cells are lysed as per the center sample. This TRIzol-cell lysate is retained to serve as the ‘edge’ sample.

As a frugal alternative, we present here a ‘low tech’ moderate throughput means of isolating cells at the migrating edge from cells distal to this edge in an in vitro 2D wound assay. This is accomplished through the design and fit of a novel ‘collar’ device to an existing and commercially available fence-barrier device originally designed for migration assay (Fig. 1A-B).^16^ Specifically, herein are reported instructions for fabrication and implementation of the collar followed by proof of principle results derived from qRT-PCR analysis of primary epidermal human keratinocytes at the edge of a migrating, in vitro, two-dimensional monolayer.

## Results and discussion

The original fence-barrier migration assay works essentially by forming a temporary inner-well within the existing well of a 24-well tissue culture plate, initially confining cells added to this inner-well from leaking or migrating into the surrounding well of the culture ware. Standard procedure for this migration assay involves seeding cells into the inner-well formed by the fence-barrier (from here on referred to as the ‘fence’) then allowing these cells to attach to the well bottom. The fence is removed after cell attachment releasing the adherent cell monolayer, which can now be observed as it migrates and/or proliferates into unoccupied surfaces (Fig. 1C, i-iii).

In adaptation of this protocol a ‘collar’ device was designed and fitted to the fence that enables the fence to be returned to its original position within the culture ware, reforming the inner-well. The collar is necessary to increase the fence circumference to exactly fit the well, eliminating lateral displacement of the fence in the outer-well during removal and replacement. This adaptation to the procedure allows the fence to act as a ‘hole-punch’ that bisects the cell monolayer (Fig. 1C, iv), with cells that have expanded beyond the fence perimeter separated from cells within these original bounds. Thus, a subset of cells that comprises the unwounded central cell population can be harvested from inside the fence perimeter with an appropriate protocol (Fig. 1C, v). In this example TRIzol was used for the cell lysis and extraction of RNA. After aspirating the centre cell lysate from the inner-well, edge cells were similarly lysed and collected from outside the fence to obtain the cell population that comprises the leading edge (Fig. 1C, vi).

**Figure 2.**
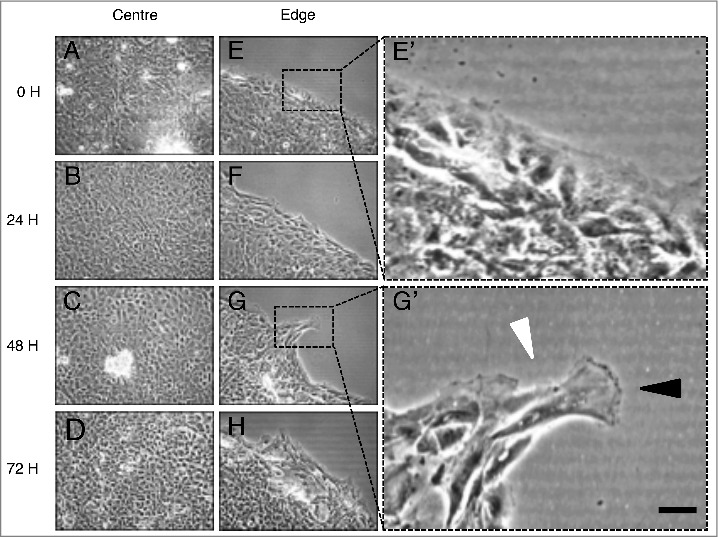
Phase contrast light micrographs of keratinocyte monolayer centers and edges. (A-D) Monolayer centers at 0, 24, 48 and 72 hours of migration. (E-H) Monolayer edges at 0, 24, 48 and 72 hours of migration. (E’) Inset of (E) monolayer edge at 0 hours of migration. (G’) Inset of (G) after 48 hours of migration. The black arrowhead points toward extending lamellipodia. The white arrowhead indicates the spindle morphology of a migratory keratinocyte. Scale bar represents 10 µm.

It is recommended that the seeding density and attachment conditions be optimised for each cell type. In the example reported here, primary human keratinocytes at passage 2 were seeded at a density of 50,000 cells per well and allowed to attach for 24 hours. In this study migration periods of 24, 48 and 72 hours were chosen. The edge of the monolayer was observed with phase contrast light microscopy 0, 24, 48 and 72 hours after removing the fence (Fig. 2.). After 0 hours of migration the morphology of cells at the edge of the monolayer was similar to cells at the monolayer center (Fig. 2E, 2E'). Over time in culture, cells at the monolayer edge display migratory characteristics such as the extension of lamellipodia and irregular positioning (Fig. 2G, 2G’).

**Figure 3.**
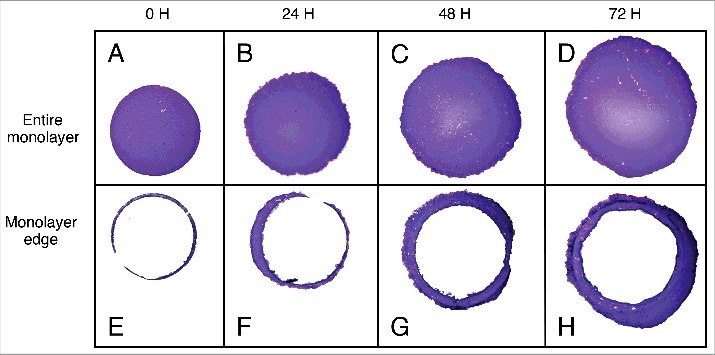
Monolayers stained with crystal violet before and after harvesting the monolayer centers A-D) Entire monolayers at 0, 24, 48 and 72 (left to right) hours of migration. E-G) Remaining cells after the center of the monolayers have been harvested with TRIzol at 0, 24, 48 and 72 (left to right) hours of migration. Images were taken at 3x magnification.

To demonstrate the collection of leading edge and central cell populations and as detailed in the methods (and illustrated in Fig. 1C, v), TRIzol was introduced into the inner-well to lyse and collect the central cell population. However, instead of proceeding to collect remaining keratinocytes of the monolayer edge, as for RNA extraction (below), edge keratinocytes were fixed within the culture well with 4% paraformaldehyde, stained with crystal violet and imaged with a stereo dissection microscope (Fig. 3E-H). In parallel, formaldehyde fixation and crystal violet staining was also performed for intact keratinocyte monolayers i.e. without removing the central monolayer (Fig. 3A-D). Resultant images at 0 hours yielded a circular monolayer with a defined edge, in line with phase contrast light microscopy data (Fig. 3E'). An attempt to bisect the monolayer at 0 hours of migration is also depicted (Fig. 3E). However it was discovered that replacement of the fence at this time point crushed the edge keratinocytes and did not permit collection of an edge fraction - thus all 0 hour samples for gene expression analysis outlined below consist of the RNA extracted from the entire 0 hour monolayer without subdivision. Over 24, 48 and 72 hours of culture keratinocyte monolayers increased in diameter (Fig. 3B-D) and proportionally, so too did the population of cells comprising the edge (Fig. 3F-H).

**Figure 4.**
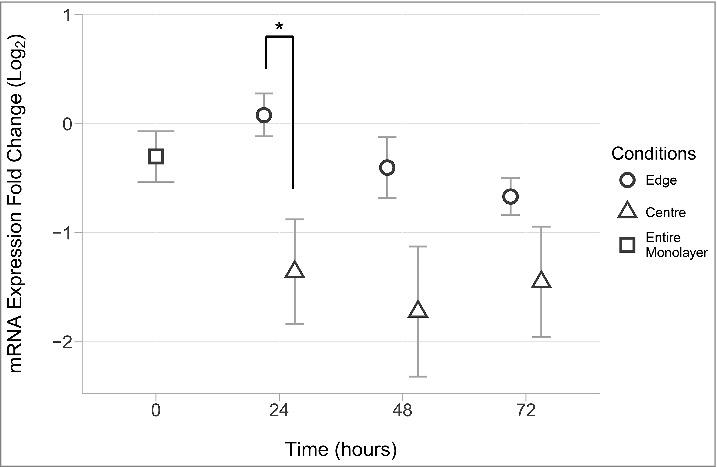
A comparison of center and edge Dll4 mRNA expression. After primary human keratinocytes were seeded for 24 hours, RNA was extracted from edge and center samples as described. Briefly, after expansion of the monolayer for 24, 48 or 72 hours, fences were returned to their respective wells and TRIzol was injected into the recreated inner well, lysing cells and capturing RNA from the monolayer center. The lysate and fence were removed and an equal volume of TRIzol was added to the culture well to lyse and capture the remaining monolayer edge. RNA was then precipitated and used to create cDNA for qRT-PCR analysis. Presented are the mean (n = 7) fold changes in ΔCτ values for Dll4 mRNA expression at 0, 24, 48 and 72 hours post migration. All ΔCτ values for edge (circles) and center (triangles) conditions were normalized to the mean Dll4 expression in the entire monolayer at 0 hours (square) and Log_2_ transformed. Error bars show the standard error of the mean. The asterisk ‘*’ indicates a P value < 0.05. Pairwise t-tests were performed comparing the edge and center for each time point and corrected for multiple testing (Bonferroni).

To assess whether RNA extracted from the edge and center cell subsets obtained in our model reflected gene expression signatures of migrating keratinocytes reported in the literature, RNA was precipitated from the TRIzol-cell lysates and assayed for mRNA expression of delta-like ligand 4 (Dll4). High Dll4 mRNA expression is reported to be confined to keratinocytes at the immediate edge of a migrating sheet in vitro, as observed with mRNA in situ hybridization.^10^ Thus, assay of Dll4 provides a means of validating the experimental procedure presented here. After RNA qualification and quantification, cDNA was generated for qRT-PCR. RT-PCR was performed using oligonucleotide primers designed to detect Dll4 mRNA using the Δ∁τ method to compare Dll4 expression between the cell populations from the leading edge and center after 24, 48 and 72 hours of migration (Fig. 4). Concurrent with the expression of Dll4 mRNA reported by Riahi and colleagues, the mean Dll4 expression was found to be significantly greater in keratinocytes harvested from the leading edge than from keratinocytes harvested from the monolayer center, after 24 hours of migration (p < 0.05).^10^ The elevated expression observed after 24 hours was no longer evident after 48 and 72 hours of migration. This was not surprising considering that Riahi and colleagues' analysis of Dll4 expression used single cell gene expression analysis. By employing in situ hybridization techniques Riahi and co-authors discovered that Dll4 expression was upregulated, but strictly confined to leader cells, that comprise a small percentage of the migrating front.

While it is unlikely that keratinocytes at the leading edge down-regulate their expression of Dll4, the reduction in difference between edge and center samples at 48 and 72 hours may be explained by the dilution of cells at the leading edge. This is likely caused by cells behind the leading edge that populate the zone between the leading edge and the cells harvested as the center sample, but are not exposed to free space. As the monolayer expands, this sub-population of cells dilutes the true edge cells, decreasing their contribution to the transcriptome of the total cell population captured as edge sample.

It is important to keep in mind that relatively small numbers of leader cells occupy the leading edge. This and the effect of dilution both considered, our model still yielded data indicating an increased expression of Dll4 mRNA in the edge sample that is concurrent with the restriction of Dll4 expression described in the literature.^10^ From this perspective our results provide a proof of principle and a demonstration of the sensitivity of the technique.

This effect of sample dilution due to unstimulated cells was previously addressed in work published by Lan and colleagues.^14^ Briefly, they have designed a stamp etched with multi-parallel ridges and valleys that when pressed into a confluent cell monolayer creates many thin strips or ‘islands’ of cells significantly increasing the proportion of cells responding to free space in culture. However, as noted by the authors, while this procedure is useful for increasing the cell numbers that display a regenerative response to wounding, it is only applicable to cells that exhibit a firm attachment to the culture substrate, with cells that more firmly attach to one another demonstrating “a tendency to lift off the dish.” In our experience with similar multi-parallel wounding devices, keratinocytes exhibit this tendency to remain cohesive in preference to adhesive with the culture substratum. An elegant solution to this problem was devised by J.K. Klarlund.^15^ Klarlund describes the deposition of many agarose droplets on the flask bottom, before cell seeding. Once seeded, cells would grow around the agarose droplets, which were then digested with agarase. This gentle method of inducing extensive free-space stimulus has not been utilised for RNA extraction, nonetheless one can speculate that this would be simple to achieve and require a separate culture of unwounded cells as a control. In comparison to these models, the fence and collar design presented here does not create a wound to the same extent as that developed by Lan et al., but to our knowledge, is unaffected by variations in the adhesive strength of different cell types. Nor does our model supply the amount of wound edge reported by Klarlund, without significant upscaling. To its advantage, however, our model includes an internal subset of control cells, which may prove an important difference. In our model control cells comprise the expanding monolayer, but do not comprise the edge itself. Thus, our model may capture signaling exchanged between edge-stimulated and edge-distal cells, which could be compromised when separating wounded and control cell cultures.

One may question whether cells incur damage as the fence is removed from the monolayer, generating inconsistent migratory stimuli at the monolayer edge. In this regard, previous research indicates that using similar silicone-based cell barriers, especially compared with scratch or crush migration assays, circumvents cell damage to the constrained monolayer edge.^17^ Damage incurred by cells during replacement of the fence in preparation for RNA extraction is unlikely to induce artificial RNA or protein expression changes as the procedure is performed at low temperatures (on ice) and quickly (over 2–3 minutes) precluding signal mechanotransduction from having any major effect on the harvested transcriptome. However, phosphorylation changes that occur quickly, in some cases less than 0.3 seconds at physiological temperatures, may not be slowed sufficiently by chilling to avoid inducing artifacts during fence replacement.^18^

## Conclusions

The method reported here describes a cost effective modification of an existing migration assay that enables explorative investigation of the molecular changes that occur at the leading edge of collectively migrating cells. Moreover, while not investigated here, this method may be extended to investigate changes in the expression of proteins or non-coding RNAs, or such responses induced by leading edge cells encountering culture surfaces prepared with one or more proteins of interest. Thus this simple adaptation adds a new dimension to an established method in the investigation of wound healing and collective cell migration in vitro.

## Materials and methods

### Cell culture

Primary keratinocytes were isolated from surgical skin discards samples as described previously under approval from both the hospitals and Queensland University of Technology (QUT), Brisbane, Australia (QUT ethics approval number 130000003).^19^ In brief, the skin discards were digested in 0.125% trypsin EDTA (Gibco, pH 7.2–8) at 4°C overnight. The dermis was then removed from the epidermis with sterile forceps and the papillary dermis was gently scraped to remove cells and collected by centrifugation at 100 × g for 5 min. Keratinocytes were then seeded onto twice irradiated (25 Gy; Australian Red Cross Blood Service, Brisbane, QLD, Australia) murine fibroblast 3T3 cells (ATCC, Manassas, VA, USA ; i3T3s) in Green's medium at a density of $1\;\times \;10^{6}$ cells per T75 tissue culture flask.^20^ Green's medium comprised Dulbecco's modified Eagle's medium with 25 mM glucose (Invitrogen, Grand Island, NY, USA) and HAMS-F12 (Invitrogen) in a 3:1 ratio, supplemented with 2 mmol L^−1^ L-glutamine, 1000 IU mL^−1^ penicillin / streptomycin, 0.01% (v / v) non-essential amino acids (all Invitrogen), 0.2 µmol L^−1^ triiodothyronine, 180 µmol L^−1^ adenine, 0.1 µg mL^−1^ cholera toxin, 0.4 µg mL^−1^ hydrocortisone, 5 µg mL^−1^ transferrin (all Sigma-Aldrich, Castle Hill, NSW, Australia), 10% foetal calf serum (FCS; Thermo Scientific HyClone, Waltham, MA, USA), 10 ng mL^−1^ epidermal growth factor (Invitrogen) and 1 µg mL^−1^ insulin (Sigma-Aldrich). Upon keratinocytes reaching 80% confluence, i3T3 cells were removed from the co-culture by briefly washing cells with 0.05% trypsin EDTA (Gibco, pH 7.2–8). Under these conditions keratinocytes remain firmly attached to the culture flask and were removed by secondary incubation (5 min) in trypsin EDTA at 37°C and gentle agitation of the flask. Keratinocytes were harvested, counted, and 50,000 cells transferred into the inner-wells of the fences (Aix Scientifics, Aachen, Germany) and allowed to attach for 24 hours.^16^

### Collar fabrication

Collars were designed using Solidworks 2014 Premium Edition (Dassault Systèmes, France) and cut in house from a sheet of 3 mm thick polytetrafluoroethylene using the ILS12.75 D platform with ‘Laser Interface+’ (Universal Laser Systems, USA) control software.

### Application of fence-collar system

After keratinocyte attachment, before removing any fences, the bottom of the culture plates were inspected by eye for media that may have leaked from the inner-wells into the surrounding outer-wells during the seeding and incubation stages. The positions of wells that displayed any evidence of a leak were recorded. Subsequently, the fence-collar units were removed from the culture well using sterile forceps. In wells where leaks were observed the well was inspected using a phase contrast light microscope for any cell attachment outside the bounds of the inner-well or any inconsistencies present in the monolayer edge. In the case that such irregularities were observed offending wells were omitted from further experimentation. Remaining monolayers that required further incubation were washed with warm DMEM to remove un-attached cells. Then 750 µL of Green's media was added to each well and the monolayers were returned to incubation. After 24, 48, or 72 hours of incubation each monolayer was washed with DMEM. To ensure that damage to the monolayer incurred when replacing the fence did not alter the monolayer gene expression, 250 µl of RNALater® (Sigma-Aldrich) as an RNA preservative was added to each well before the fence was returned. To lyse the inner cell subset, RNALater was aspirated from each well and the monolayers were washed briefly with PBS. The culture plate was placed on ice and the fence-collar units were returned to their respective wells. Forceps were used to apply gentle downward pressure on the fence, ensuring an adequate seal between the fence and the well bottom. Following this, 200 µl of TRIzol was pipetted into the inner-well and pipetted vigorously up and down to lyse the cells (for at least 15 repetitions). The TRIzol-cell lysate was then aspirated from the inner-well and retained. A second volume of TRIzol was injected and in a similar fashion, pipetted vigorously, aspirated and added to the first volume of lysate; a total sample volume of 400 µl TRIzol-cell lysate. This sample was termed the ‘center’ sample. The fence-collar unit was then removed from the outer-well. At this stage the well was inspected for any TRIzol that had leaked from the center well, contaminating the edge cell sample. This was discernable by eye as any volume of TRIzol wetting the remaining edge cells. In the case of Leaked TRIzol the effected samples were discarded. Having inspected the well, 400 µl of TRIzol was pipetted over the remaining adherent keratinocytes - that could be observed by eye as a faint ring. This volume of TRIzol-cell lysate was aspirated and retained as the ‘edge’ sample.

### RNA extraction and qRT-PCR

RNA was precipitated from the TRIzol-cell lysate as per the manufacturer's recommendations and resuspended in RNAse-free water (Invitrogen). The Agilent 2100 bioanalyzer was used in conjunction with an RNA6000 nano chip (Agilent) to assess RNA concentrations and integrity. Only RNA with an RNA integrity number (RIN) greater than 7 was used to make cDNA, as per the company recommendations. For each sample, 500ng of RNA was reverse transcribed, in 10 µL reactions, prepared in respect to the Superscript III first strand cDNA synthesis kit (Invitrogen) instructions. Primers were designed using Primer-BLAST to amplify the Dll4 gene Fwd: 5′ CAACCCTCTCCAACTGCCCTTCAATTT, Rvs: 3′ GCGATCTTGCTGATGAGTGCATCT and RPL32 Fwd: 5′ GATCTTGATGCCCAACATTGGTTATG, Rvs: 3′ GCACTTCCAGCTCCTTGACG.^21^ Dll4 and RPL32 cDNA levels were assayed by qRT-PCR in 10 µL reactions containing 10 ng of cDNA template, 5 µL of 2x SYBR Green RT-PCR master mix and forward and reverse primers at a concentration of 20 nM, using a QuantStudio™ 7 Flex Real-Time PCR System in 384-well format.

### Analysis and statistics

Dll4 expression was calculated using the Δ∁τ method and relative to RPL32 expression as an endogenous house keeping control. An average was calculated of Δ∁τ for samples at 0 hours of migration (n = 7), that was used as a normalization factor. The Δ∁τ of Dll4 at each time point (including 0 hours) for the edge and center conditions was divided by the mean of Δ∁τ for 0 hours. The Δ∁τ values were converted to fold changes by raising 2 to the power of −Δ∁τ then transformed to log base 2. Statistical analyses of mean Dll4 expression differences were performed in R employing three pairwise t-tests and correcting p-values for multiple testing using the Bonferroni method of correction for multiple comparisons.^22,23^

## Abbreviations

DNA: deoxyribose nucleic acid
Dll4: delta-like ligand 4
DMEM: Dulbecco's modified Eagle's medium
ECM: extracellular matrix
EDTA: ethylenediaminetetraacetic acid
LAM: laser assisted microdissection
qRT-PCR: quantitative reverse transcription polymerase chain reaction
RNA: ribose nucleic acid
RPL32: ribosomal protein L32
i3T3: irradiated murine fibroblast 3T3 cells
